# Characterization of the Phenanthrene-Degrading *Sphingobium yanoikuyae* SJTF8 in Heavy Metal Co-Existing Liquid Medium and Analysis of Its Metabolic Pathway

**DOI:** 10.3390/microorganisms8060946

**Published:** 2020-06-23

**Authors:** Chong Yin, Weiliang Xiong, Hua Qiu, Wanli Peng, Zixin Deng, Shuangjun Lin, Rubing Liang

**Affiliations:** State Key Laboratory of Microbial Metabolism, Joint International Research Laboratory of Metabolic and Developmental Sciences, School of Life Sciences and Biotechnology, Shanghai Jiao Tong University, Shanghai 200240, China; yching@sjtu.edu.cn (C.Y.); stogqy@gmail.com (W.X.); qh1997@sjtu.edu.cn (H.Q.); chinapengwl@163.com (W.P.); zxdeng@sjtu.edu.cn (Z.D.); linsj@sjtu.edu.cn (S.L.)

**Keywords:** polycyclic aromatic hydrocarbons, *Sphingobium yanoikuyae* SJTF8, phenanthrene, heavy metal, bioremediation, tolerance

## Abstract

Polycyclic aromatic hydrocarbons (PAHs) are common organic pollutants with great carcinogenic threaten, and metal/PAH-contaminated environments represent one of the most difficult remedial challenges. In this work, *Sphingobium yanoikuyae* SJTF8 was isolated and identified with great and stable PAH-degrading efficiency even under stress conditions. It could utilize typical PAHs (naphthalene, phenanthrene, and anthracene) and heterocyclic and halogenated aromatic compounds (dibenzothiophene and 9-bromophenanthrene) as the sole carbon source. It could degrade over 98% of 500 mg/L phenanthrene in 4 days, and the *cis*-3,4-dihydrophenanthrene-3,4-diol was the first-step intermediate. Notably, strain SJTF8 showed great tolerance to heavy metals and acidic pH. Supplements of 0.30 mM of Cu^2+^, 1.15 mM of Zn^2+^, and 0.01 mM of Cd^2+^ had little effect on its cell growth and phenanthrene degradation; phenanthrene of 250 mg/L could still be degraded completely in 48 h. Further, the whole genome sequence of *S. yanoikuyae* SJTF8 was obtained, and three plasmids were found. The potential genes participating in stress-tolerance and PAH-degradation were annotated and were found mostly distributed in plasmids 1 and 2. Elimination of plasmid 2 resulted in the loss of the PAH-degradation ability. On the basis of genome mining results, the possible degrading pathway and the metabolites of *S. yanoikuyae* SJTF8 to phenanthrene were predicted.

## 1. Introduction

Pollution caused by aromatic hydrocarbons is a matter of great concern worldwide because of its abundant distribution and toxic effects on the environment [1,2,3]. Sixteen polycyclic aromatic hydrocarbons (PAHs) are already identified as priority pollutants by the United States Environmental Protection Agency (US EPA) [1]. Microbial biodegradation can decompose harmful organic pollutants into nontoxic or low-toxicity products and are safe, economic, and applied easily [4,5]. Many genera of Gram-positive and -negative bacteria, fungi, and algae have been found able to degrade PAHs [6,7,8,9,10,11,12,13]. In aerobic bacteria, the first step degradation of various PAHs has been proved to be the oxidation of arenes catalyzed by a multicomponent dioxygenase, generating the corresponding *cis*-dihydrodiol, which is subsequently dehydrogenated by dehydrogenase and rearomatized to the diol intermediates [14,15]. These diol intermediates may then be cleaved by intradiol or extradiol ring-cleaving dioxygenases through either an *ortho*-cleavage pathway or *meta*-cleavage pathway, leading to intermediates like salicylate and/or protocatechuate and ultimately converting to tricarboxylic acid (TCA) cycle intermediates [14,16]. Despite many PAH-degrading microorganisms having been found, many critical aspects of their metabolism such as the dispersion and evolution of degrading genes, the intermediate metabolites, the degradation pathway, and the regulatory mechanisms are still not very clear.

Among the PAH-degrading bacteria, strains from the sphingomonads (composed of *Sphingomonas*, *Sphingobium*, *Sphingopyxis*, and *Novosphingobium*) showed great degradation efficiency and environment suitability [17]. *Sphingomonas* sp. CHY-1 was found to be capable of degrading chrysene, naphthalene, phenanthrene, and anthracene [11]. *Sphingobium yanoikuyae* B1 can utilize biphenyl, naphthalene, phenanthrene, and toluene as the sole carbon source [13]. Dioxygenase, salicylate 1-hydroxylase, and *cis*-dihydrodiol dehydrogenase involved in PAHs degradation in strain CHY-1 were characterized, and the crystal structure of the dioxygenase was revealed [18,19,20,21,22,23]. Nevertheless, the elucidation on the genetic mechanisms of PAHs degradation by sphingomonads was with very little progress, as the catabolic genes responsible for PAHs in sphingomonads was not in cluster distribution like those in other non-sphingomonads like *Pseudomonas*, *Burkholderia*, and *Rhodococcus* [17].

On the other hand, the combined pollution of organic contaminants and heavy metals has become a major concern and represents a high percentage of the hazardous waste on the priority lists for remediation [24,25]. Many heavy metals, such as Cd, Cr, Cu, Hg, Ni, Pb, and Zn, together with PAHs often accumulate in sediments due to the effects of industrial, municipal, and mining activities [26]. Heavy metals can affect PAHs degradation by changing the surface properties of microbes and by interfering with enzymes of microbes [27]. Co-existing heavy metals can prolong the bacterial adaptation periods and can reduce the biodegradation rates [28]. Cd, Cu, Hg, and Cr have significant effects on the degradation efficiency of 2-chlorophenol, 3-chlorobenzoate, phenol, and benzoate by anaerobic bacterial consortia [29]. Cu and Cd reduced the degradation of phenanthrene by *Sphingobium* sp. PHE-SPH and *Ochrobactrum* sp. PHE-OCH [30]. However, many studies were focused on the degradation rate and tolerance ability of strains in the combined pollutants; the genome basis of organics metabolism and metal resistance in microorganisms are still needed.

In this study, strain SJTF8 isolated from PAH-contaminated soil was found with great phenanthrene-degrading efficiency and heavy-metal resistance, and its degradation stability in the heavy-metal-PAH co-existing conditions were detected. Moreover, the whole genome sequence of this strain was obtained and genome analysis revealed the presence of several genes involved in PAH degradation and heavy metal resistance in genome and plasmid. Further, the degrading pathway and the metabolites of this strain to phenanthrene were predicted.

## 2. Materials and Methods

### 2.1. Cultures and Chemicals

M9 minimal medium (Na_2_HPO_4_ 6.0 g, KH_2_PO_4_ 3.0 g, NaCl 0.5 g, NH_4_Cl 1.0 g, CaCl_2_ 0.01 g, FeCl_3_ 0.002 g, and MgCl_2_ 0.25 g/L, pH 7.2) was used for the enrichment and isolation of PAH-degrading strains. Luria–Bertani medium (LB, tryptone 10.0 g, yeast extract 5.0 g, and NaCl 8.0 g/L, pH 7.2) was used for strain culture. Solid plates were prepared by supplying agar (15.0 g/L) into liquid medium. Different aromatic chemicals (phenanthrene, naphthalene, anthracene, pyrene, dibenzothiophene, 9-bromophenanthrene, benz[*a*]anthracene, benz[*b*]anthracene, catechol, salicylic acid, pathalic acid, protocatechuate, xylene, and biphenyl, > 99% purity, HPLC grade) were purchased from Sigma-Aldrich (Allentown, PA, USA). HPLC-grade methanol and acetonitrile (> 99.5% purity) were purchased from EMD (Gibbstown, NJ, USA). All of the other chemicals were of analytical grade.

### 2.2. Enrichment and Isolation of Phenanthrene-Degrading Strains

The soil samples used for strain enrichment were collected from Shanghai, China (no specific permissions were required, and this work did not involve any endangered or protected species). Twenty grams of soil sample were inoculated into 200 mL of M9 medium containing 250 mg/L phenanthrene and cultured in a shaker with 180 r/min at 30 °C until the medium changed from transparent to obvious yellow due to the generation of 2′-hydroxy muconic semialdehyde, a degradation product of phenanthrene [31]. Then, a 20 mL culture was transferred into a fresh 200 mL M9 medium containing 250 mg/L phenanthrene and cultured as above. After three to five rounds of enrichment, the culture was diluted and spread onto M9 plate with 250 mg/L of phenanthrene. After three-day cultivation at 30 °C, the positive colonies were picked and the phenanthrene-utilizing efficiency were detected (described below). The strain with a high removal rate for phenanthrene was named SJTF8 and was deposited in China General Microbiological Culture Collection Center (CGMCC).

### 2.3. The Physiological and Evolutionary Analysis of Strain SJTF8

The isolated strain SJTF8 was characterized based on its morphological, physiological, and biochemical properties according to the Bergey’s Manual of Determinative Bacteriology [32]. The evolutionary status of strain SJTF8 was analyzed by constructing the phylogenetic tree based on the 16S rDNA sequence. A single colony of strain SJTF8 was inoculated into LB medium and cultured at 30 °C overnight. The genomic DNA was extracted with TIANamp Bacteria DNA Kit (Tiangen Biotech Co., Beijing, China), and the 16S rDNA gene was amplified using the Bacterial 16S rDNA Kit (TaKaRa Biotechnology Co., Dalian, China) according to the manufacturer’s instructions. The PCR procedure was performed with denaturing at 94 °C for 5 min, 30 repeated times of denaturizing at 94 °C for 30 s, annealing at 55 °C for 30 s, elongating at 72 °C for 1.5 min, and further elongating at 72 °C for 5 min. The PCR fragment was sequenced by Personalbio Co., Ltd. (Shanghai, China), and the 16S rDNA sequence of strain SJTF8 was deposited into GenBank under accession number MH179331.1. The nearest relatives of strain SJTF8 were obtained by BLAST analysis based on the 16S rDNA gene sequence in the National Center for Biotechnology Information (NCBI). The phylogenetic tree was constructed with MEGA 7.0 by the neighbor joining method with 1000 replications. The evolutionary distances were calculated with the Kimura two-parameter distance model.

### 2.4. Utilization Detection of Phenanthrene and Other Aromatics by Strain SJTF8

A single colony of strain SJTF8 was inoculated into 50 mL LB broth and cultured on a rotary shaker with 180 r/min at 30 °C to OD_600_ about 0.5. Cells were harvested, washed three times with sterilized water, and resuspended with M9 medium. The cell inoculum was inoculated into 1 L fresh M9 medium with phenanthrene of different concentrations (50, 250, and 500 mg/L) to a final concentration of OD_600_ about 0.05. The flasks without chemicals and the flasks without cell inoculum were used as blanks to assess the abiotic loss. All flasks were cultured at 30 °C in the shaker (200 r/min) for 15 days. Every 12 or 24 h, 5 mL cultures were taken out to detect the cell density and the phenanthrene residues. The degradation efficiency of strain SJTF8 to other aromatic chemicals (naphthalene, anthracene, pyrene, dibenzothiophene, 9-bromophenanthrene, benz[*a*]anthracene, benz[*b*]anthracene, catechol, salicylic acid, pathalic acid, protocatechuate, xylene, and biphenyl) were detected in the same process.

### 2.5. Quantification and Metabolites Detection of Phenanthrene Degraded by Strain SJTF8

A reversed-phase HPLC system was used for the detection and quantification of phenanthrene and other aromatic chemicals. The phenanthrene-degrading efficiency of strain SJTF8 was determined according to the loss of phenanthrene. Briefly, 3 mL of cell cultures were supplied with equal volume of acetonitrile and then filtered through 0.22-μm filters (Millipore, USA). Ten microliters were injected into the HPLC detection system (Agilent 1260 Infinity LC, USA). The setting of HPLC/UV was as follow: ZORBAX Eclipse Plus C18 column (5 μm, 4.6 × 150 mm, Agilent, USA) was used as the solid phase, and the mobile phase was the gradient mixture of water and methanol (v/v): 0.00–3.00 min 50.0/50.0, 15.00–17.00 min 10.0/90.0, and 18.00–20.00 min 50.0/50.0. The column temperature was 30 °C, the flow rate was set at 1 mL/min, and the UV wavelength was 254 nm. The quantities in culture were calculated with the standard curve of each chemical. The *R^2^* values for all standard curves were > 0.99. All data were the average values of five independent experiments at each time point with standard errors. The degradation efficiency of strain SJTF8 to other aromatic chemicals (listed in 2.4) was detected and calculated similarly.

The metabolite of phenanthrene degraded by strain SJTF8 was analyzed on the UPLC (Ultra Performance Liquid Chromatography) system (Waters Acquity system, Waters, MA, USA) coupled to an electrospray ionization (ESI)-mass spectrometer. Separation was achieved on a reversed-phase C18 column (Acquity UPLC BEH C18; 1.7 μm; 100 × 2.1 mm; Waters, MA, USA) with a flow rate of 0.4 mL/min at 30 °C. The injection volume was 10 μL; the mobile phase comprised the gradient mixture of water and acetonitrile (v/v): 0.00–3.00 min 50.0/50.0, 25.00–28.00 min 0.0/100.0, and 28.10–30.00 min 50.0/50.0. Mass spectral data were collected in ESI mode in separate runs on a Waters HDMS-QTOF synapt GI mass spectrometer operating in a scan mode of 50–900 m/z. The capillary voltage was set at 3000 V; the source and desolvation temperatures were 80 °C and 250 °C, respectively. The cone gas flowrate was 50 L/h. The predicted elemental composition of individual intermediate was calculated on MassLynx Mass Spectrometry Software (Waters, MA, USA).

### 2.6. Effect of Heavy Metals and Other Stress Conditions on the Cell Growth and the Phenanthrene-Degrading Efficiency of Strain SJTF8

To test the tolerance of strain SJTF8 to heavy metals, the cell growth of strain SJTF8 were detected. Stock solutions (10 g/L) of CuCl_2_·2H_2_O, CdCl_2_·2.5H_2_O, and ZnCl_2_ in sterilized distilled water were prepared. Different heavy metals were added in M9 medium containing 2% glucose: Cu^2+^ (0.15, 0.30, 0.59, and 1.18 mM), Cd^2+^ (0.01, 0.02, 0.04, and 0.08 mM), or Zn^2+^ (0.29, 0.57, 1.15, and 2.29 mM). Exponentially growing cells were used, the cell densities were determined every two hours by a fully automatic growth curve analyzer, and the growth curves were plotted. The growth inhibition plots were calculated by comparing the cell growth in cultures added with various concentrations of Cu^2+^, Cd^2+^, and Zn^2+^ and those in cultures without heavy metals supplements, and the EC_50_ (Effective concentrations for 50% growth inhibition) were calculated by GraphPad Prism 7. The phenanthrene degradation efficiency of strain SJTF8 under different conditions were investigated in the M9 medium using phenanthrene (250 mg/L) as the sole carbon source, supplied with Cu^2+^ (0.15, 0.30, 0.59, or 1.18 mM), Cd^2+^ (0.01, 0.02, 0.04, or 0.08 mM), Zn^2+^ (0.29, 0.57, 1.15, or 2.29 mM), or designated pH (5.0–9.0). The blank control was the group without cell inoculation. The strain culture and the HPLC detection of phenanthrene residues were the same as above. Three parallels were set for each sample, and five independent experiments were performed for each condition; the average values were calculated with standard errors.

### 2.7. Plasmid Elimination of Strain SJTF8

The plasmid elimination was performed by high-temperature culture method [33]. A single colony of strain SJTF8 was streaked and cultured on LB solid medium plates at 35 °C, keeping a single colony re-lined onto a fresh LB plate every 24 h. After three generations of culture, several colonies were picked out to detect the efficiency of plasmid elimination by PCR amplification with specific primers (Table S2).

### 2.8. Whole Genome Sequencing and Genome Mining of Strain SJTF8

The genomic DNA of strain SJTF8 was extracted with the QIAamp DNA Mini Kit (Qiagen, CA), and two libraries were constructed. One was a 10.0 kb insert SMRT-bell library sequenced on the PacBio RS II platform using two single-molecule real-time (SMRT) cells by Pacific Biosciences (PacBio) RS II sequencer (Pacific Biosciences, CA, US). Another one was a 400 bp insert library, sequenced with paired-end sequencing mode (PE250) using Illumina Miseq platform. The gaps were closed by specific PCR and Sanger sequencing. The data of PacBio RS II platform were assembled using the HGAP4 and CANU version 1.6 [34,35], and the data of Illumina Miseq platform were corrected with Kmer and assembled with A5-miseq and SPAdes genome assembler [36,37]. All the contigs were analyzed by MUMmer 3.0 [38], and the sequences were corrected by Pilon [39]. The genome sequence was annotated using the Rapid Annotation using Subsystem Technology (RAST) database [40]. The complete genome sequence of *S. yanoikuyae* SJTF8 was submitted to NCBI under the accession numbers NZ_CP033230.1, NZ_CP033227.1, NZ_CP033228.1, and NZ_CP033229.1. The coding sequences (CDSs) were predicted using Glimmer 3.0 [41], and gene functions were annotated with the NCBI-NR [42], evolutionary genealogy of genes: Non-supervised Orthologous Groups (eggNOG) [43], Swiss-Prot [44], and Kyoto Encyclopedia of Genes and Genomes (KEGG) databases [45]. The average nucleotide identity (ANI) values were assessed using JSpeciesWS website (http://jspecies.ribohost.com/jspeciesws) [46]. Briefly, the genome sequence of strain SJTF8 was uploaded and fifteen strains representing the most closely related genera were selected for the pairwise genome comparison on the basis of BLAST and MUMmer to calculate the whole genome identity.

## 3. Results

### 3.1. Isolation and Identification of Sphingobium yanoikuyae SJTF8

A new bacterium strain SJTF8 with phenanthrene-degrading capability was enriched and isolated from the soil. It was short-rod shaped and Gram-negative; the colonies appeared light yellow and opaque. Biochemical analysis showed that it could utilize sodium citrate, catechol, salicylic acid, and protocatechuate. Strain SJTF8 was deposited into the CGMCC with the accession number CGMCC15632. The taxonomic analysis of strain SJTF8 showed that it was close to *Sphingobium yanoikuyae* (Figure S1). The 16S rDNA sequence of strain SJTF8 had 99.78% similarity with that of strain *S. yanoikuyae* KUDC1818 and 100% similarity with strain *S. yanoikuyae* ANT3D. After completing the whole genome sequencing of strain SJTF8, ANI analysis was also performed to compare the genome similarity of fifteen close relatives. The ANIm values of the genome of strain SJTF8 were compared with those of *S. yanoikuyae* ATCC 51230 and *S. yanoikuyae* B1 were 96.3% and 99.4%, and the ANIb values were 95.1% and 98.4%, respectively (Table S1). Therefore, strain SJTF8 was classified to *Sphingobium yanoikuyae*.

### 3.2. S. yanoikuyae SJTF8 Could Degrade Phenanthrene and Several Other Typical Aromatics Efficiently

**Figure 1:**
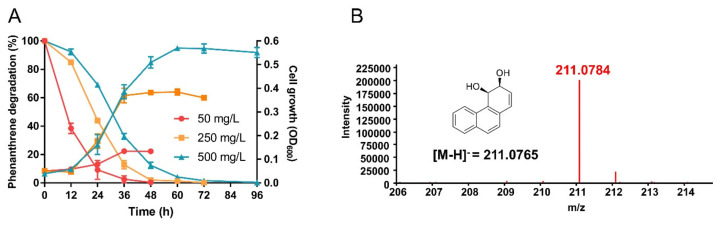
The phenanthrene-degrading efficiency and product of strain SJTF8: (**A**) The biodegradation efficiency and growth curves of *S. yanoikuyae* SJTF8. Cells were cultured in M9 medium with phenanthrene (50–500 mg/L) as the sole carbon source, and samples were detected every 12 h. The residual phenanthrene in cultures were determined by HPLC system, and the growth curves were also plotted. Error bars represented the standard errors of five independent experiments. (**B**) The UPLC/MS result of the intermediate in phenanthrene biodegradation: In M9 medium containing 50 mg/L phenanthrene, samples were taken after 12 h of cultivation. The peak was analyzed to be consistent with *cis*-3,4-dihydrophenanthrene-3,4-diol.

The degradation efficiency of *S. yanoikuyae* SJTF8 to phenanthrene and several other aromatic chemicals (naphthalene, anthracene, pyrene, dibenzothiophene, 9-bromophenanthrene, benz[*a*]anthracene, benz[*b*]anthracene, catechol, salicylic acid, pathalic acid, protocatechuate, xylene, and biphenyl) was investigated by culturing strain SJTF8 with these chemicals as the sole carbon source and detected the cell growth and their residues in culture. Growth curves and the HPLC results showed that *S. yanoikuyae* SJTF8 exhibited efficient phenanthrene-degrading capability, and its cell growth was positively correlated with the phenanthrene degradation (Figure 1A). When 50 mg/L of phenanthrene was used, the degradation efficiency of 97.3% was observed after 36-h culture, and after 48 h, over 99.0% of phenanthrene in culture was degraded. Meanwhile, the 36-h degradation efficiencies of this strain to 250 mg/L and 500 mg/L of phenanthrene were 87.2% and 67.2%; after 60 h and 72 h, this chemical in that concentration was degraded completely (Figure 1A). The degradation efficiency and period varied according to the phenanthrene concentration, possibly due to the potential response effect or limited speed of intermediates. Further UPLC/MS analysis confirmed that, at the early stage, a large amount of *cis*-3,4-dihydrophenanthrene-3,4-diol could be detected in the culture with phenanthrene, probably the first-step intermediate of phenanthrene by strain SJTF8 (Figure 1B). Compared with other reported phenanthrene-degrading strains, strain SJTF8 showed a stronger degradation efficiency [9,12,47].

Furthermore, the degradation of strain SJTF8 to other typical aromatic chemicals were detected. Results indicated that strain SJTF8 could also utilize naphthalene, anthracene, catechol, salicylic acid, protocatechuate, biphenyl, and dibenzothiophene as the sole carbon source efficiently. High degradation efficiency to the halogenated PAH (9-bromophenanthrene) and the heterocyclic PAH (dibenzothiophene) were also observed (Table 1). However, it was hard for strain SJTF8 to degrade pathalic acid and xylene, and the high-molecular-weight PAHs are composed of four benzene rings, such as benz[*a*]anthracene, benz[*b*]anthracene, and pyrene (Table 1). During the culture process, color change of the culture was observed frequently due to the compound structure change or the metabolite accumulation [48]. All these results demonstrated that strain SJTF8 had a broad substrate spectrum and could efficiently degrade typical PAHs.

### 3.3. S. yanoikuyae SJTF8 Could Tolerate High Concentration of Heavy Metals and Degrade Phenanthrene Efficiently

Heavy metals are one of the common co-pollutants existing in PAH pollution environments which can influence the cell survival greatly, cause a serious shift of the microorganism community, and result in the great reduction of bioremediation efficiency. Cu and Zn are the two heavy metals with the highest levels in some PAH-polluted conditions [26]. Cd has been grouped as a potent human carcinogenic metal by the International Agency for Research on Cancer and has been accepted as one of indicators for heavy metal contamination [49,50].

**Figure 2:**
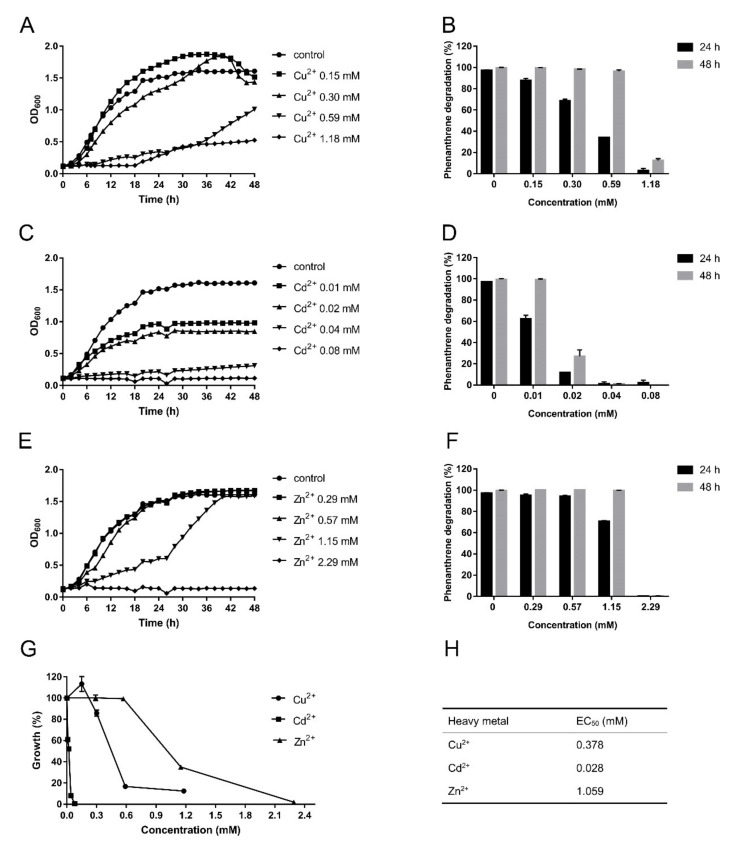
Effect of heavy metals on the cell growth and phenanthrene degradation of *S. yanoikuyae* SJTF8: (**A**) *S. yanoikuyae* SJTF8 was cultured in M9 medium with glucose and Cu^2+^ (0 to 1.18 mM). (**B**) *S. yanoikuyae* SJTF8 was cultured in M9 medium with phenanthrene (250 mg/L) and Cu^2+^ (0 to 1.18 mM). The concentration of phenanthrene in culture was detected with the HPLC system at 24 h and 48 h, and the degradation efficiency was calculated. Error bars represent standard errors of five tests. (**C**) *S. yanoikuyae* SJTF8 was cultured in M9 medium with glucose and Cd^2+^ (0 to 0.08 mM). (**D**) *S. yanoikuyae* SJTF8 was cultured in M9 medium with phenanthrene (250 mg/L) and Cd^2+^ (0 to 0.08 mM). The concentration of phenanthrene in culture was detected with HPLC system at 24 h and 48 h, and the degradation efficiency was calculated. Error bars represent standard errors of five tests. (**E**) *S. yanoikuyae* SJTF8 was cultured in M9 medium with glucose and Zn^2+^ (0 to 2.29 mM). (**F**) *S. yanoikuyae* SJTF8 was cultured in M9 medium with phenanthrene (250 mg/L) and Zn^2+^ (0 to 2.29 mM). The concentration of phenanthrene in culture was detected with the HPLC system at 24 h and 48 h, and the degradation efficiency was calculated. Error bars represent standard errors of five tests. (**G**) Growth inhibition plots for Cu^2+^, Cd^2+^, and Zn^2+^ of *S. yanoikuyae* SJTF8 cultured after 24 h in M9 medium with glucose containing various heavy metals of different concentrations. (**H**) The EC_50_ of heavy metals to *S. yanoikuyae* SJTF8 was calculated by GraphPad Prism 7.

To investigate the tolerance of strain SJTF8 to heavy metals and to determine the effects of co-existed heavy metals on its phenanthrene-degrading efficiency, the three heavy metals (Cu, Cd, and Zn) were supplied into the culture, respectively. Results showed that the strain SJTF8 exhibited great tolerance to the three heavy metals, although its tolerance to cadmium was relatively lower than the other two. It grew normally under Cu^2+^ and Zn^2+^ (0.30 and 0.57 mM) stress, while low concentration of Cd^2+^ (0.04 mM) inhibited cell growth significantly in the M9 liquid medium (Figure 2A,C,E,G). The degradation of strain SJTF8 to phenanthrene under the heavy metal stress showed similar tendency (Figure 2B,D,F). In the M9 medium with phenanthrene (250 mg/L) as the sole carbon source and Cu^2+^ supplied in 0.30 mM, over 68% of phenanthrene was degraded in 24 h and almost all the phenanthrene was degraded at 48 h (Figure 2B). Adding Zn^2+^ of 0.57 mM into the M9 medium with phenanthrene had no significant effect on the degradation to this chemical; even in the culture containing Zn^2+^ of 1.15 mM, strain SJTF8 still degraded about 70% of phenanthrene at 24 h and degraded it completely at 48 h (Figure 2F). Although strain SJTF8 showed a relatively low tolerance to cadmium, considering the higher toxicity of cadmium, it is still of great importance for aquatic system restoration in the real environment. When Cd^2+^ of 0.01 mM was used, a certain inhibitory effect on cell growth was observed. However, at this concentration, the biodegradation efficiency to phenanthrene was still at about 62% at 24 h and all the chemicals could be degraded at 48 h (Figure 2D).

The relative growth inhibition curves showed that Cd^2+^ had the strongest inhibition to SJTF8 and that Zn^2+^ had the weakest inhibition (Figure 2G). The EC_50_ of *S. yanoikuyae* SJTF8 towards Cu^2+^, Cd^2+^, and Zn^2+^ were 0.378, 0.028, and 1.059 mM, respectively (Figure 2H). As the concentrations of heavy metals in real environments were far lower than those used in the experiments, *S. yanoikuyae* SJTF8 had great heavy-metal tolerance and may be useful for the biodegradation in the combined polluted environments. In addition, the suitability and degrading stability of *S. yanoikuyae* SJTF8 to different pHs were also detected. The results indicated that *S. yanoikuyae* SJTF8 exhibited a stable adaptability to the relative acidic conditions; under pH 5.0, it still maintained over 50% degradation efficiency to phenanthrene of 250 mg/L at 48 h (Figure S2). In a word, *S. yanoikuyae* SJTF8 exerted great and broad tolerance to heavy metals and relative acidic conditions and retained a stable phenanthrene-degradation efficiency.

### 3.4. Whole Genome Sequence Analysis of S. yanoikuyae SJTF8

**Figure 3:**
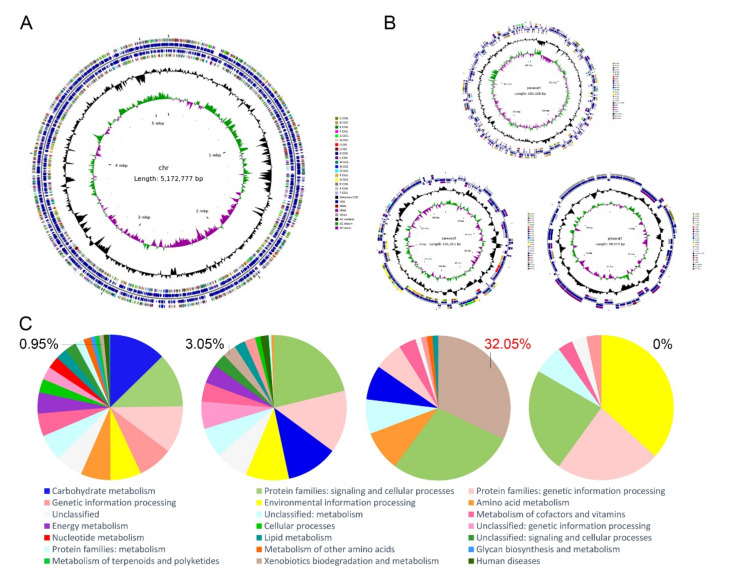
The circular maps and functional category analysis of *S. yanoikuyae* SJTF8 chromosome and plasmids DNA: The circular maps of chromosome (**A**) and the three plasmids (**B**) of *S. yanoikuyae* SJTF8 are shown. From the inside to outside: (I) sequence scale mark; (II) GC skew; (III) GC content; (IV, VII) Clusters of Orthologous Group (COG) for each Coding Sequence (CDS); and (V, VI) the position of CDS, tRNA, and rRNA on the genome. (**C**) The functional categories analysis of *S. yanoikuyae* SJTF8 was based on BlastKOALA in Kyoto Encyclopedia of Genes and Genomes (KEGG). The percentage represented the ratio of xenobiotics (such as polycyclic aromatic hydrocarbons (PAHs)) biodegradation and metabolism genes in chromosome DNA and plasmids 1–3 of *S. yanoikuyae* SJTF8.

To gain the genetic basis of phenanthrene degradation and environment adaptability, the whole genome of *S. yanoikuyae* SJTF8 was sequenced. The complete genome of *S. yanoikuyae* SJTF8 consists of 5,172,777 bp of chromosomal DNA with GC content of 64.31%. Three plasmids were detected in this strain (base pairs and GC contents: 505,328 bp (62.37%), 191,911 bp (62.65%), 59,573 bp (61.34%)) (Figure 3A,B and Table 2). A total of 4896 genes (4781 protein-coding genes) were annotated in the chromosome, including 12 rRNAs, 62 tRNAs, and 25 ncRNAs; 547 genes (545 protein-coding genes), 188 genes (186 protein-coding genes), and 72 genes (72 protein-coding genes) were found in the three plasmids (Table 2).

The predicted CDSs were further classified into 21 Clusters of Orthologous Groups (COGs) of proteins categories based on the COG function classification (Table 3). The six most abundant groups were group S (1065 Open Reading Frames (ORFs), function unknown), group L (280 ORFs, replication, recombination, and repair), group E (258 ORFs, amino acid transport and metabolism), group C (257 ORFs, energy production and conversion), group M (250 ORFs, cell wall/membrane/envelope biogenesis), and group P (249 ORFs, inorganic ion transport and metabolism). Gene alignment and annotation showed that the percentage of the xenobiotics (such as PAHs) biodegradation and metabolism genes in chromosome and the three plasmids of *S. yanoikuyae* SJTF8 were 0.95%, 3.05%, 32.05% and 0% (Figure 3C). It meant that the potential degradation genes for PAHs may distribute in the genome, especially the plasmid 2.

### 3.5. Genes in Plasmid 2 Were Responsible for PAH Degradation

**Figure 4:**
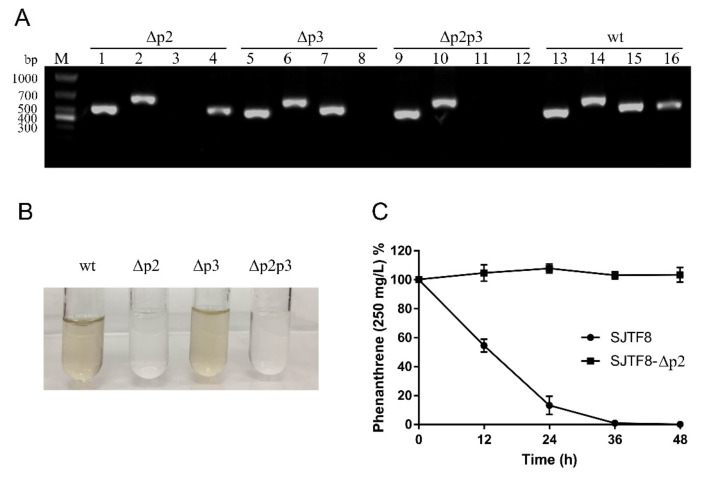
The degradation efficiency of *S. yanoikuyae* SJTF8 and its derivate strains without plasmids: (**A**) PCR detection of wild-type *S. yanoikuyae* SJTF8 and the three derivate strains without plasmid 2 and/or 3 wt was the wild-type strain SJTF8; Δp2, Δp3, and Δp2p3 represented the strain without plasmid 2, the strain without plasmid 3, and the strain without plasmid 2/3, respectively. 1/5/9/13 were the PCR products amplified from the chromosome; 2/6/10/14 were the PCR products amplified from the plasmid 1; 3/7/11/15 were the PCR products amplified from the plasmid 2; and 4/8/12/16 were the PCR products amplified from the plasmid 3. (**B**) Culture color detection of *S. yanoikuyae* SJTF8 and the derivate strains without plasmids 2 and/or 3. wt was the wild-type strain SJTF8; Δp2, Δp3, and Δp2p3 represented the strain without plasmid 2, the strain without plasmid 3, and the strain without plasmid 2/3, respectively. (**C**) The detection of the phenanthrene degradation efficiency of *S. yanoikuyae* SJTF8 and the derivate strains without plasmids 2 (SJTF8-Δp2): The strains were cultured in M9 medium with phenanthrene of 250 mg/L for 2 days. The concentration of phenanthrene in culture was detected with HPLC system every 12 h, and the degradation efficiency were calculated. Error bars represent standard errors of five tests.

Genes involved in the bacterial resistance of heavy metals or degradation of xenobiotics are often found in plasmid DNA [51]. To determine the metabolic genes responsible for PAH degradation, plasmid elimination was performed. For *S. yanoikuyae* SJTF8, plasmid 2 and/or plasmid 3 were eliminated easily while plasmid 1 was difficult to be eliminated, implying the presence of essential genes for cell growth and metabolism. When plasmid 2 was eliminated, the new strain with other two plasmids (plasmids 1 and 3) lost the phenanthrene-degrading capability (Figure 4B,C). However, the strain without plasmid 3 could still retain its phenanthrene degradation efficiency. This meant that the catabolic genes for PAHs was not in plasmid 3. Also, the degradation properties of strain with only plasmid 1 was the same as that of the strain without plasmid 2 (Figure 4B). These results demonstrated that plasmid 2 was closely related to the PAHs degradation in *S. yanoikuyae* SJTF8.

### 3.6. The Phenanthrene Catabolic Pathway in S. yanoikuyae SJTF8

**Figure 5:**
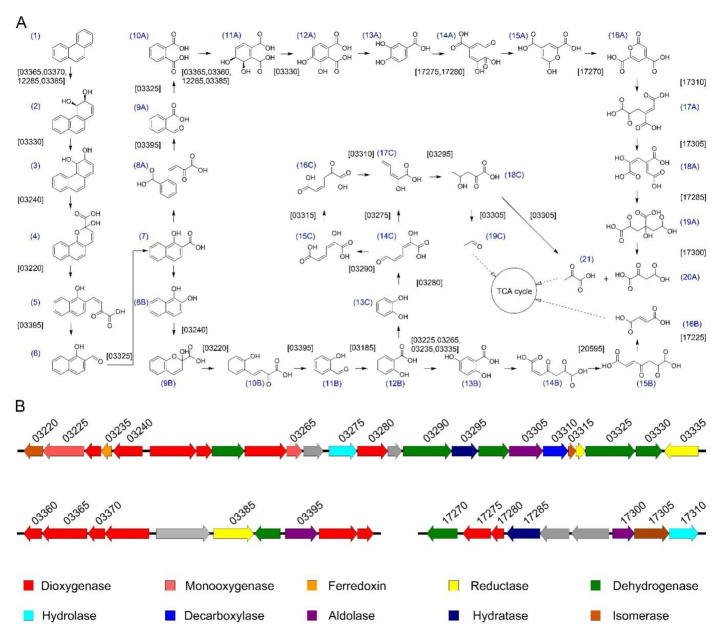
Proposed phenanthrene catabolic pathway in *S. yanoikuyae* SJTF8 and the organization of genes involved in the catabolic pathway: (**A**) Proposed phenanthrene catabolic pathway of strain SJTF8. Compound names: (1) phenanthrene, (2) (3S,4R)-3,4-dihydrophenanthrene-3,4-diol, (3) phenanthrene-3,4-diol, (4) 2-hydroxy-2H-benzo[*h*]chromene-2-carboxylic acid, (5) (Z)-4-(1-hydroxynaphthalen-2-yl)-2-oxobut-3-enoic acid, (6) 1-hydroxy-2-naphthaldehyde, (7) 1-hydroxy-2-naphthoic acid, (8A) (Z)-2-(3-carboxy-3-oxoprop-1-en-1-yl)benzoic acid, (9A) 2-formylbenzoic acid, (10A) phthalic acid, (11A) (5S,6R)-5,6-dihydroxycyclohexa-1,3-diene-1,2-dicarboxylic acid, (12A) 3,4-dihydroxyphthalic acid, (13A) 3,4-dihydroxybenzoic acid, (14A) (2E,4E)-2-hydroxy-4-(2-oxoethylidene)pent-2-enedioic acid, (15A) 2-hydroxy-2H-pyran-4,6-dicarboxylic acid, (16A) 2-oxo-2H-pyran-4,6-dicarboxylic acid, (17A) (E)-4-oxobut-1-ene-1,2,4-tricarboxylic acid, (18A) (1Z,3E)-4-hydroxybuta-1,3-diene-1,2,4-tricarboxylic acid, (19A) 2-hydroxy-4-oxobutane-1,2,4-tricarboxylic acid, (20A) 2-oxosuccinic acid, (8B) naphthalene-1,2-diol, (9B) 2-hydroxy-2H-chromene-2-carboxylic acid, (10B) (E)-4-(2-hydroxyphenyl)-2-oxobut-3-enoic acid, (11B) 2-hydroxybenzaldehyde, (12B) 2-hydroxybenzoic acid, (13B) 2,5-dihydroxybenzoic acid, (14B) (Z)-4,6-dioxohept-2-enedioic acid, (15B (E)-4,6-dioxohept-2-enedioic acid, (16B) fumaric acid, (13C) pyrocatechol, (14C) (2E,4Z)-2-hydroxy-6-oxohexa-2,4-dienoic acid, (15C) (2E,4Z)-2-hydroxyhexa-2,4-dienedioic acid, (16C) (Z)-5-oxohex-2-enedioic acid, (17C) (E)-2-hydroxypenta-2,4-dienoic acid, (18C) 4-hydroxy-2-oxopentanoic acid, (19C) acetaldehyde, and (21) 2-oxopropanoic acid. (**B**) Organization of the genes involved in phenanthrene catabolism in strain SJTF8: Enzymes associated with phenanthrene degradation were indicated by different colors, and gray color indicated other enzymes excluding the shown ten enzyme types.

To study the PAH-degrading genes in *S. yanoikuyae* SJTF8, deep genome analysis was performed based on the genome sequence and the KEGG database. Results revealed that at least 41 genes potentially involved in the degradation of phenanthrene were found and degradation pathway was predicted (Table S3). Mineralization, co-metabolic transformation, and oxidation are the three different modes of microbial degradation of PAHs by pure bacterial cultures [15]. In the aerobic degradation of PAHs, the favored mechanism by sphingomonads is oxidation [15]. The first step of phenanthrene aerobic degradation often begins with the oxidation of dioxygenase acting on single donors with incorporation of two atoms of molecular oxygen to the aromatic ring generating the *cis*-dihydrodiol [1,14]. Dehydrogenase catalyzes the dehydrogenation of *cis*-dihydrodiol to phenanthrene-3,4-diol. Then, a ring cleavage is completed by the catalysis of dioxygenase and isomerase. The compound 1-hydroxy-2-naphthoic acid, a common intermediate of PAH metabolism, can be obtained through the catalysis of aldolase and dehydrogenase. 1-Hydroxy-2-naphthoic acid is further degraded mainly by two metabolic pathways; one is the *o*-phthalic acid pathway (Figure 5A, compounds 8A–21), and the other is the salicylic acid pathway (Figure 5A, compounds 8B–16B and 8B–19C). The salicylic acid is then further processed through the gentisate (Figure 5A, compounds 13B–16B) or catechol pathways (Figure 5A, compounds 13C–19C). Then, these products are metabolized to tricarboxylic acid cycle intermediates. Sphingomonads tend to utilize the salicylic acid pathway (*Sphingomonas* sp. strain KH3-2) or a combination of both salicylic acid and *o*-phthalic acid pathways in phenanthrene degradation (*N. pentaromativorans* strain US6-1) but do not use the *o*-phthalic acid pathway alone [17]. As *S. yanoikuyae* SJTF8 utilized salicylic acid as the sole carbon source rather than *o*-phthalic acid, the salicylic acid pathway was the proposed pathway in *S. yanoikuyae* SJTF8 (Table 1, Figure 5A, compounds 8B–16B and 8B–19C). Further analysis indicated that the genes encoding enzymes responsible for PAH metabolism in strain SJTF8 mainly distributed in plasmid 2 and existed in clusters; a few homologue genes were also found in the chromosome (Figure 5B and Table S3).

In addition, 36 genes encoding proteins responsible for heavy metal resistance, such as genes encoding enzymes of periplasmic heavy metal sensor and heavy metal efflux transporter, were found in the genome of *S. yanoikuyae* SJTF8. Among these 36 genes, 14 genes were in the chromosome, 18 genes were in plasmid 1, 4 genes were in plasmid 3, and none were in plasmid 2. (Table S4). For example, genes *copB*, *copC*, and *copD* were found in the chromosome and plasmid 1 of strain SJTF8 (Table S4). These are the three structural genes of *cop* operon which support the hypothesis that Cu^2+^ resistance is due to either periplasmic binding or extracellular sequestration [52]. Cadmium/copper-translocating P-type ATPase were also found in plasmid 1 and function as the efflux pumps that export Cd^2+^ or Cu^2+^ from the cell interior (Table S4) [52]. The CzcA family heavy metal efflux resistance nodulation cell division (RND) transporter is essential for cation transport to form a membrane tunnel (Table S4) [52]. The *czc* system is an efflux system that removes Zn^2+^ and Cd^2+^ [52]. In addition to the resistance genes responsible for copper, cadmium, and zinc, resistance genes for mercury and arsenic had also been discovered (Table S4). This may be the great tolerance basis for *S. yanoikuyae* SJTF8 to heavy metals.

## 4. Discussion

PAH is an environmental pollutant that has received widespread attention for a long time. Among the many methods for degrading PAHs, microbial remediation is considered an ecological, economical, and safe method for treating PAHs pollution [31]. Although many bacteria capable of degrading PAHs have been isolated, strains that can degrade PAHs with high efficiency, broad substrate spectrum, and great adaptability for stress are still expected. Understanding the metabolic pathways and molecular mechanisms of PAH biodegradation is gradually deepening. In this study, *S. yanoikuyae* SJTF8 was proven to be able to utilize the typical aromatic chemicals as the sole carbon source efficiently and to degrade more than 98% of 500 mg/L phenanthrene in 4 days. Compared with other reported sphingomonads, it showed excellent degradation characteristics. For example, *Sphingobium* sp. FB3 degraded 99% of phenanthrene (100 mg/L) in 10 days, *Sphingobium yanoikuyae* LD29 could degrade 92% of phenanthrene (50 mg/L) in 4 days, and *Sphingomonas* sp. 1-21 degraded 98% of phenanthrene (200 mg/L) after 10 days of incubation [53]. *Novosphingobium pentaromativorans* US6-1 degraded 86.62% of phenanthrene (10 mg/L) within 24 h, and *Sphingomonas koreensis* ASU-06 degraded 98.6% of phenanthrene (100 mg/L) in 15 days [17]. Therefore, strain SJTF8 showed the fastest degradation to phenanthrene in the reported sphingomonads.

In a real environment, the contamination of aromatic compounds appeared with a mixture of multiple low-molecular-weight and high-molecular-weight aromatic compounds. Therefore, strains with broad substrate spectrum were suitable for the actual environment remediation. The phenanthrene-degrading strain *Pseudomonas stutzeri* ZP2 could grow with phenanthrene or naphthalene as the sole carbon source but could not degrade anthracene [47]. Sphingomonads were considered as the excellent degraders of aromatics. A strain of *Sphingobium yanoikuyae* was isolated as a degrader of the monocyclic aromatic compound ibuprofen [54]. *Sphingomonas* sp. GY2B could utilize naphthalene, phenanthrene, 1-hydroxy-2-naphthoic acid, 2-naphthol, and phenol as the sole carbon sourc, except pyrene [55]. Similarly, strain SJTF8 could degrade the monocyclic aromatics like salicylic acid, catechol, and protocatechuate and the classic PAHs like naphthalene, phenanthrene, and anthracene efficiently but could not degrade the high-molecular-weight aromatic compounds such as pyrene and benz[*a*]anthracene. However, strain SJTF8 could also utilize the heterocyclic aromatic compounds like dibenzothiophene and the halogenated aromatic compounds like 9-bromophenanthrene as the sole carbon source, expanding its application potential of this strain.

In addition, the combined pollution of PAHs with other pollutants like heavy metals exists extensively in actual environments. Indeed, more than 67% of the contaminated sites contain both the organic pollutants and heavy metals [1,2,3]. High concentrations of metals can damage cell membranes, can inactivate enzymes, and can damage DNA [30,52]. As the co-existing factors interfere with the degradation process and cell growth greatly, biodegradation of PAHs in metal-contaminated environments is complicated and of great concern. In this work, *S. yanoikuyae* SJTF8 showed high tolerance to Cu^2+^, Cd^2+^, and Zn^2+^ of high concentrations and maintained efficient PAH-degrading capability in the co-existing culture, although the degradation period was prolonged to some extent. Gram-negative *Pseudomonas* sp. ORNaP2 was isolated in the presence of naphthalene as the sole carbon source showing excellent tolerance to heavy metals, and the EC_50_ of strain ORNaP2 towards Cu^2+^, Cd^2+^, and Zn^2+^ were 0.90, 0.03, and 0.30 mM, respectively [56], while the EC_50_ of *S. yanoikuyae* SJTF8 to the three metals were 0.378, 0.028, and 1.059 mM, respectively. Herein, strain SJTF8 showed a higher tolerance towards Zn^2+^, whereas it was more sensitive towards Cu^2+^ and Cd^2+^ than strain ORNaP2. However, it showed much more tolerance to these heavy metals and more stable degradation efficiency than those of other sphingomonads. Even in the presence of 0.01 mM of Cd^2+^, the degradation efficiency of strain SJTF8 to phenanthrene can still achieve over 99% in 2 days. *Stenotrophomonas maltophilia* degraded about 30% and 45% of benz[*a*]pyrene at 48 h and at 7 days in the presence of 10 mg/L (0.16 mM) Cu^2+^ [57]. With 0.5 mg/L (0.004 mM) of Cd^2+^, the removal ratio of phenanthrene by *S. xenophagum* D43FB reduced from about 80% to 20% [31]. Thus, strain SJTF8 showed great adaptability in liquid medium and high PAH-degrading efficiency, implying its appliance potential in real environments. Meanwhile, metal inhibition of biodegradation could be reduced by introducing metal-tolerant genes in the organic-degrading microorganisms [29,58]. In the genome of strain SJTF8, 36 metal-tolerance related genes have been found, which may be the genetic basis of the great resistance to heavy metals of this strain.

Following the isolation of many microorganisms with degradation capabilities to aromatic compounds, their genomes have been sequenced and the key enzymes for initial degradation-like dioxygenase have been identified [13,31,48,52]. However, despite the rare sequenced sphingomonads, the catabolic genes for individual degradative pathways in sphingomonads are also very difficult to be arranged because of their scattered distribution in plasmids or chromosome, different from the cluster distribution in non-sphingomonads [15]. In this work, the genome sequence of *S. yanoikuyae* SJTF8 was obtained and the genes potentially involved in PAHs degradation were annotated. Most of the PAH-degrading genes were found in the plasmid 2 of this strain and in several clusters. For *Pseudomonas*, the structure of catabolic operons of pND6-1 in *Pseudomonas* sp. strain ND6 is similar to that of pDTG1 in *P. putida* NCIB 9816-4 and plasmid NAH7 in *P. putida* G7. One “upper pathway” operon (genes for the conversion of naphthalene to salicylate) and one “lower pathway” operon (genes for the conversion of the intermediates in the salicylate to tricarboxylic acid cycle of the *meta*-cleavage pathway) are arranged on the plasmid [59]. Similarly to the structure of the gene arrangement in sphingomonads (*Novosphingobium aromaticivorans* F199 [60], *Sphingobium yanoikuyae* B1 [61], *Sphingomonas* sp. strain CHY-1 [30], *Sphingobium xenophagum* D43FB [31], *Sphingobium* sp. strain P2 [62]), these catabolic genes in SJTF8 are not ordered in their sequential enzymatic reactions. The genes for “upper” and “lower” pathways are encoded in all encountered PAH-degrading operons. The peculiar gene organization shows an evolutionary deviation from the non-sphingomonads, making these sphingomonads a very particular group among the PAH degraders [14]. The “flexible” gene organization could be one of the mechanisms that allows sphingomonads to adapt quickly and efficiently to novel compounds in the environment [28]. Therefore, genome sequencing and mining of strain SJTF8 can help better understand the whole process of PAH metabolism and can provide support to further study the degradation mechanism of PAHs.

## 5. Conclusions

In conclusion, this work identified the PAH-degrading *S. yanoikuyae* SJTF8 and explored its degradation characteristics to different aromatic chemicals in liquid medium. This strain showed a broad substrate spectrum of typical PAHs and great tolerance to the widely existing heavy metals. Whole genome sequences analysis revealed the genetic basis of this strain in PAH degradation and environment suitability. Most of the PAH-degrading genes was proven to be located in plasmid 2, and its metabolic pathway for phenanthrene was predicted. This study can enrich the PAH-degrading strains pool, can help the understanding of PAH biodegradation under heavy metal stresses, and can promote bioremediation appliance for the real polluted environments.

## Figures and Tables

**Table 1:** Degradation efficiency of strain SJTF8 to aromatic compounds.

Aromatic Chemicals	Degradation Capability ^a^	Degradation Efficiency (%) ^b^	Medium Color
Naphthalene	+	> 99	persistent brown
Dibenzothiophene	+	70	temporary red
9-Bromophenanthrene	+	54	persistent brown
Anthracene	+	33	temporary pink
Benz[*a*]anthracene	–	–	–
Benz[*b*]anthracene	–	–	–
Pyrene	–	–	–
Catechol	+	> 99	persistent brown
Salicylic acid	+	> 99	colorless
Phthalic acid	–	–	–
Protocatechuate	+	> 99	colorless
Xylene	–	–	–
Biphenyl	+	> 99	persistent brown

^a^ Strain SJTF8 was cultured in M9 medium with different aromatic chemicals of 50 mg/L as the sole carbon source for 2 days, and the cell growth were determined. When pyrene, benz[*a*]anthracene, and benz[*b*]anthracene were used, the culture periods were 7 days. ^b^ The degradation efficiency of strain SJTF8 to different aromatic chemicals was detected by HPLC system at the end of the 2-day or 7-day cultures.

**Table 2:** General features of *S. yanoikuyae* SJTF8 genome.

Genome Features	Chromosome	Plasmid 1	Plasmid 2	Plasmid 3
Size (bp)	5,172,777	505,328	191,911	59,573
GC content (%)	64.31%	62.37%	62.65%	61.34%
Total genes	4,896	547	188	72
Coding genes	4781	545	186	72
rRNA	12	0	0	0
tRNA	62	0	0	0
Other ncRNA	25	2	2	0

**Table 3:** COG functional categories of *S. yanoikuyae* SJTF8 genome.

COG Categories	Categories Function	ORF Number	Value %
A	RNA processing and modification	0	0
B	Chromatin structure and dynamics	0	0
C	Energy production and conversion	257	5.249
D	Cell cycle control, cell division, chromosome partitioning	31	0.633
E	Amino acid transport and metabolism	258	5.270
F	Nucleotide transport and metabolism	66	1.348
G	Carbohydrate transport and metabolism	241	4.922
H	Coenzyme transport and metabolism	117	2.390
I	Lipid transport and metabolism	165	3.370
J	Translation, ribosomal structure and biogenesis	159	3.248
K	Transcription	248	5.065
L	Replication, recombination and repair	280	5.719
M	Cell wall/membrane/envelope biogenesis	250	5.106
N	Cell motility	32	0.654
O	Posttranslational modification, protein turnover, chaperones	156	3.186
P	Inorganic ion transport and metabolism	249	5.086
Q	Secondary metabolites biosynthesis, transport and catabolism	99	2.022
R	General function prediction only	0	0
S	Function unknown	1065	21.752
T	Signal transduction mechanisms	193	3.942
U	Intracellular trafficking, secretion, and vesicular transport	86	1.757
V	Defense mechanisms	69	1.409
W	Extracellular structures	0	0
Y	Nuclear structure	0	0
Z	Cytoskeleton	0	0
–	Not in eggNOG	875	17.872

## Supplementary Materials

The following are available online at https://www.mdpi.com/2076-2607/8/6/946/s1, Table S1: The ANI values between strain SJTF8 and other fifteen strains, Table S2: The list of oligonucleotides used in this work, Table S3: Genes related to aromatic compound metabolism in the genome of *S. yanoikuyae* SJTF8, Table S4: Genes related to heavy metal resistance in the genome of *S. yanoikuyae* SJTF8, Figure S1: The Phylogenetic tree of strain SJTF8, Figure S2: Effect of pH on the phenanthrene degradation of *S. yanoikuyae* SJTF8.
