# Supplementary material for: Characterization of the Phenanthrene-Degrading Sphingobium yanoikuyae SJTF8 in Heavy Metal Co-Existing Liquid Medium and Analysis of Its Metabolic Pathway
Source: Microorganisms. 2020 Jun 23;8(6):946. doi: 10.3390/microorganisms8060946 (PMC7355620; doi:10.3390/microorganisms8060946)
Supplement: Supplementary file 1 [file microorganisms-08-00946-s001.pdf]

Supplementary Materials

Characterization of the Phenanthrene-Degrading *Sphingobium yanoikuyae* SJTF8 in Heavy Metal Co-Existing Liquid Medium and Analysis of Its Metabolic Pathway

Chong Yin[1], Weiliang Xiong[1], Hua Qiu, Wanli Peng[1], Zixin Deng[1], Shuangjun Lin[1], Rubing Liang[1]*

[1] State Key Laboratory of Microbial Metabolism, Joint International Research Laboratory of Metabolic & Developmental Sciences, School of Life Sciences and Biotechnology, Shanghai Jiao Tong University, 800 Dongchuan Road, Shanghai 200240, China

* Corresponding author: Rubing Liang

State Key Laboratory of Microbial Metabolism, Joint International Research Laboratory of Metabolic & Developmental Sciences, School of Life Sciences and Biotechnology, Shanghai Jiao Tong University, 800 Dongchuan Road, Shanghai 200240, China

Tel/Fax: 86-21-34204192;

E-mail: icelike@sjtu.edu.cn

Table S1. The ANI values between strain SJTF8 and other fifteen strains.

Strains	Strain SJTF8	
	ANIb (%)	ANIm (%)
Sphingobium yanoikuyae B1	98.4	99.4
Sphingobium yanoikuyae ATCC 51230 [T]	95.1	96.3
Sphingobium czechense LL01 [T]	79.1	85.2
Sphingobium japonicum UT26S [T]	78.6	85.0
Sphingobium ummariense RL-3 [T]	78.5	84.9
Sphingobium faniae CGMCC 1.7749 [T]	77.7	84.5
Sphingomonas panacis DCY99 [T]	71.3	83.0
Sphingopyxis alaskensis RB2256 [T]	71.3	83.3
Sphingomonas aerolata NW12 [T]	71.1	83.1
Sphingopyxis macrogoltabida 203 [T]	70.9	83.5
Sphingopyxis witflariensis DSM 14551 [T]	70.9	83.2
Sphingomonas jaspsi DSM 18422 [T]	70.7	83.3
Novosphingobium rosa NBRC 15208 [T]	70.6	83.1
Novosphingobium aromaticivorans DSM 12444 [T]	70.3	83.6
Novosphingobium tardaugens NBRC 16725 [T]	69.8	83.6

[T], the typical strain of the species; ANIb, the ANI calculation based on BLAST+; ANIm, the ANI calculation based on MUMmer.

Table S2. The list of oligonucleotides used in this work.

Primer	Sequence 5′–3′	Description
chr-F	CTCGTCAACCGCTATTCCGA	Detection of the gene in chromosome DNA
chr-R	CCATCGCACAATCCGTTAGG	
p1-F	CTGGTTACGCCGCATTCAAG	Detection of the gene in plasmid 1 DNA
p1-R	TGGATACCAAACCAGACCGC	
p2-F	TTGCCGAGGATGTCGATCTG	Detection of the gene in plasmid 2 DNA
p2-R	CGAACGGGTCTGCAAGAGAT	
p3-F	GAGCAGGACCGCTATGTCTG	Detection of the gene in plasmid 3 DNA
p3-R	TCGTCGCGTAGTAGGATTGC	

Table S3. Genes related to aromatic compound metabolism in the genome of *S. yanoikuyae* SJTF8.

Location	Locus_tag	Refseq_ID	Strand	Start	End	Putative gene function
plasmid 2	EBF16_RS03185	WP_122129281.1	–	103144	104589	salicylaldehyde dehydrogenase
plasmid 2	EBF16_RS03220	WP_122129287.1	–	113942	114532	2-hydroxychromene-2-carboxylate isomerase
plasmid 2	EBF16_RS03225	WP_122129288.1	–	114529	115791	salicylate 5-hydroxylase large subunit
plasmid 2	EBF16_RS03230	WP_122129289.1	–	115816	116307	anthranilate 1,2-dioxygenase small subunit
plasmid 2	EBF16_RS03235	WP_122129290.1	–	116304	116630	naphthalene 1,2-dioxygenase ferredoxin component
plasmid 2	EBF16_RS03240	WP_122129291.1	–	116675	117574	1,2-dihydroxynaphthalene dioxygenase
plasmid 2	EBF16_RS03245	WP_122129292.1	+	117806	119224	benzoate/toluate 1,2-dioxygenase subunit alpha
plasmid 2	EBF16_RS03250	WP_122129293.1	+	119221	119715	benzoate/toluate 1,2-dioxygenase subunit beta
plasmid 2	EBF16_RS03255	WP_122129294.1	+	119699	120691	4-phospho-D-threonate 3-dehydrogenase
plasmid 2	EBF16_RS03260	WP_122129295.1	+	120702	121982	anthranilate 1,2-dioxygenase large subunit
plasmid 2	EBF16_RS03265	WP_122129296.1	+	121979	122458	salicylate 5-hydroxylase small subunit
plasmid 2	EBF16_RS03275	WP_122129298.1	+	123274	124125	2-hydroxymuconate-semialdehyde hydrolase
plasmid 2	EBF16_RS03280	WP_122129299.1	+	124144	125067	catechol 2,3-dioxygenase
plasmid 2	EBF16_RS03290	WP_011906647.1	+	125541	127025	2-hydroxymuconate-6-semialdehyde dehydrogenase
plasmid 2	EBF16_RS03295	WP_010891017.1	+	127030	127824	2-keto-4-pentenoate hydratase
plasmid 2	EBF16_RS03300	WP_010891016.1	+	127847	128785	acetaldehyde dehydrogenase
plasmid 2	EBF16_RS03305	WP_010891015.1	+	128782	129813	4-hydroxy-2-oxovalerate aldolase

plasmid 2	EBF16_RS03310	WP_010891014.1	+	129815	130585	2-oxo-3-hexenedioate decarboxylase
plasmid 2	EBF16_RS03315	WP_100868228.1	+	130587	130829	4-oxalocrotonate tautomerase
plasmid 2	EBF16_RS03320	WP_010891012.1	+	130819	131100	reductase
plasmid 2	EBF16_RS03325	WP_010891011.1	+	131120	132625	2-formylbenzoate dehydrogenase
plasmid 2	EBF16_RS03330	WP_010891010.1	+	132657	133457	*cis*-3,4-dihydrophenanthrene-3,4-diol dehydrogenase
plasmid 2	EBF16_RS03335	WP_010891009.1	–	133516	134556	naphthalene 1,2-dioxygenase ferredoxin reductase component
plasmid 2	EBF16_RS03360	WP_010891005.1	–	140103	140654	phthalate 3,4-dioxygenase subunit beta
plasmid 2	EBF16_RS03365	WP_010891004.1	–	140669	142036	PAH dioxygenase large subunit
plasmid 2	EBF16_RS03370	WP_010891003.1	–	142062	142589	PAH dioxygenase small subunit
plasmid 2	EBF16_RS03375	WP_010891002.1	–	142582	143934	ethylbenzene dioxygenase subunit alpha
plasmid 2	EBF16_RS03385	WP_010891000.1	+	145901	147127	phthalate 3,4-dioxygenase ferredoxin reductase component
plasmid 2	EBF16_RS03390	WP_010890999.1	–	147147	147935	dihydroxycyclohexadiene carboxylate dehydrogenase
plasmid 2	EBF16_RS03395	WP_010890998.1	+	148087	149073	4-(2-carboxyphenyl)-2-oxobut-3-enoate aldolase
plasmid 2	EBF16_RS03400	WP_010890997.1	+	149116	150291	anthranilate 1,2-dioxygenase large subunit
plasmid 2	EBF16_RS03405	WP_010890996.1	+	150288	150785	anthranilate 1,2-dioxygenase small subunit
chromosome	EBF16_RS12285	WP_004212347.1	–	1817218	1817409	phthalate 3,4-dioxygenase ferredoxin component
chromosome	EBF16_RS17270	WP_004208428.1	–	2881926	2882867	2-hydroxy-4-carboxymuconate semialdehyde hemiacetal dehydrogenase
chromosome	EBF16_RS17275	WP_037509214.1	–	2883027	2883875	protocatechuate 4,5-dioxygenase subunit beta
chromosome	EBF16_RS17280	WP_007708667.1	–	2883875	2884279	protocatechuate 4,5-dioxygenase

						subunit alpha
chromosome	EBF16_RS17285	WP_026109430.1	–	2884364	2885386	4-oxalomesaconate hydratase
chromosome	EBF16_RS17300	WP_037509218.1	+	2887600	2888274	4-hydroxy-4-methyl-2-oxoglutarate aldolase
chromosome	EBF16_RS17305	WP_037509220.1	+	2888267	2889331	4-oxalomesaconate tautomerase
chromosome	EBF16_RS17310	WP_037509221.1	+	2889328	2890215	2-pyrone-4,6-dicarboxylate lactonase
chromosome	EBF16_RS20595	WP_037508236.1	–	3582766	3583410	maleylpyruvate isomerase

Table S4. Genes related to heavy metal resistance in the genome of *S. yanoikuyae* SJTF8.

Location	Locus_tag	Refseq_ID	Strand	Start	End	Putative encoded function
plasmid 1	EBF16_RS00580	WP_037445067.1	+	113523	116750	CusA/CzcA family heavy metal efflux RND transporter
plasmid 1	EBF16_RS00645	WP_037445092.1	–	127397	128608	copper resistance protein B
plasmid 1	EBF16_RS00650	WP_037445095.1	–	128913	130745	copper resistance system multicopper oxidase
plasmid 1	EBF16_RS00680	WP_020819850.1	–	133716	133934	heavy-metal-associated domain-containing protein
plasmid 1	EBF16_RS00685	WP_020819851.1	+	134095	136605	copper-translocating P-type ATPase
plasmid 1	EBF16_RS00940	WP_007683374.1	–	192281	194638	heavy metal translocating P-type ATPase
plasmid 1	EBF16_RS00950	WP_020820523.1	–	195033	195977	copper homeostasis membrane protein CopD
plasmid 1	EBF16_RS00955	WP_007683372.1	–	195982	196359	copper homeostasis periplasmic binding protein CopC
plasmid 1	EBF16_RS00965	WP_007683368.1	+	196765	197205	periplasmic heavy metal sensor
plasmid 1	EBF16_RS00975	WP_007683365.1	+	197835	199760	copper resistance system multicopper oxidase
plasmid 1	EBF16_RS00980	WP_020820521.1	+	199760	200908	copper resistance protein B
plasmid 1	EBF16_RS01050	WP_007683344.1	–	214125	214928	copper resistance protein B
plasmid 1	EBF16_RS01055	WP_007683343.1	–	215108	216814	copper resistance system multicopper oxidase
plasmid 1	EBF16_RS01120	WP_007406417.1	–	225876	226355	heavy metal-responsive transcriptional regulator
plasmid 1	EBF16_RS01325	WP_004213249.1	+	261816	262145	heavy-metal-associated domain-containing protein
plasmid 1	EBF16_RS01330	WP_122129114.1	+	262225	263601	mercury (II) reductase
plasmid 1	EBF16_RS02430	WP_120252793.1	+	463386	466457	heavy metal translocating P-type ATPase

plasmid 1	EBF16_RS02465	WP_122129178.1	–	469105	471219	cadmium-translocating P-type ATPase
plasmid 2	EBF16_RS03665	WP_004213240.1	+	9372	9770	mercury transporter MerT
plasmid 2	EBF16_RS03670	WP_004213238.1	+	9798	10130	heavy-metal-associated domain-containing protein
plasmid 2	EBF16_RS03675	WP_004213237.1	+	10141	10389	mercury resistance system transport protein MerF
plasmid 2	EBF16_RS03680	WP_004213235.1	+	10392	11822	mercury (II) reductase
chromosome	EBF16_RS09255	WP_004207026.1	+	1106836	1109325	heavy metal translocating P-type ATPase
chromosome	EBF16_RS11925	WP_004212440.1	–	1739672	1740640	magnesium and cobalt transport protein CorA
chromosome	EBF16_RS17755	WP_010339676.1	–	3014091	3015143	HoxN/HupN/NixA family nickel/cobalt transporter
chromosome	EBF16_RS17770	WP_037508318.1	–	3019128	3019988	copper resistance protein B
chromosome	EBF16_RS17775	WP_037508320.1	–	3019985	3021724	copper resistance system multicopper oxidase
chromosome	EBF16_RS17785	WP_010339670.1	–	3022303	3022737	periplasmic heavy metal sensor
chromosome	EBF16_RS17795	WP_037508324.1	+	3023143	3023538	copper resistance protein CopC
chromosome	EBF16_RS18480	WP_036530299.1	+	3154515	3155255	arsenical resistance protein ArsH
chromosome	EBF16_RS18890	WP_037523027.1	–	3239828	3240574	arsenical resistance protein ArsH
chromosome	EBF16_RS19665	WP_122129904.1	–	3393083	3393829	arsenical resistance protein ArsH
chromosome	EBF16_RS19670	WP_004210521.1	–	3393826	3394167	arsenical-resistance protein
chromosome	EBF16_RS25035	WP_007012907.1	–	4505913	4506230	heavy metal-binding domain-containing protein
chromosome	EBF16_RS27150	WP_063142250.1	+	4929520	4932747	CusA/CzcA family heavy metal efflux RND transporter
chromosome	EBF16_RS27175	WP_037510720.1	+	4934777	4937272	heavy metal translocating P-type ATPase

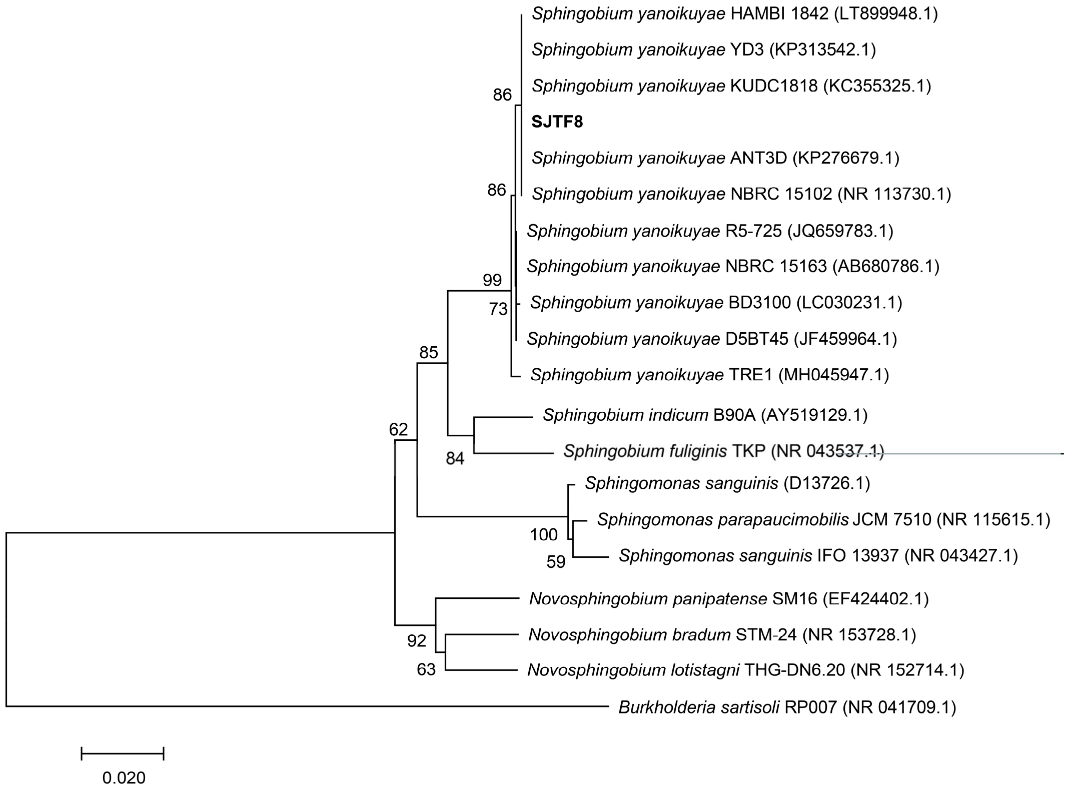

Figure S1. The Phylogenetic tree of strain SJTF8. Phylogenetic tree of the bacterial strain SJTF8 based on 16S rDNA gene sequence. The phylogenetic tree was constructed in MEGA 7.0 on the basis of the Neighbor-Joining method with kimura two-parameter model. GenBank accession numbers were indicated in parentheses and the bootstrap consensus tree was performed with 1,000 replications. The 16S rDNA gene sequence of *Burkholderia sartisoli* RP007 was used as an outgroup.

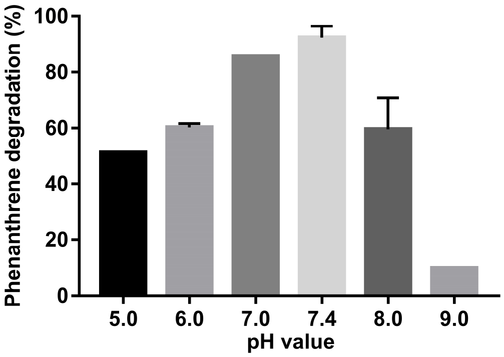

Figure S2. Effect of pH on the phenanthrene degradation of *S. yanoikuyae* SJTF8. *S. yanoikuyae* SJTF8 was cultured in M9 medium at designated pH (5.0-9.0) using phenanthrene of 250 mg/L as the sole carbon source. The concentration of phenanthrene in culture was detected with HPLC system at 48 h, and the degradation efficiency was calculated. Error bars represent standard errors of five tests.
